# A dataset from a coordinated multi-site laboratory study investigating the Hue-Heat-Hypothesis

**DOI:** 10.1038/s41597-025-05962-1

**Published:** 2025-09-19

**Authors:** Mateus Bavaresco, Roberta Jacoby Cureau, Ilaria Pigliautile, Edit Barna, Zsofia Deme Belafi, Lorenzo Belussi, Giorgia Chinazzo, Agnese Chiucchiù, Ludovico Danza, Zhipeng Deng, Bing Dong, Natasha Hansen Gapski, Liége Garlet, Veronica Martins Gnecco, Xingtong Guo, Peiman Pilehchi Ha, Hamidreza Karimian, Roberto Lamberts, Shichao Liu, Brenda da Costa Loeser, Camilla Massucci, Ana Paula Melo, Balázs Vince Nagy, Mohamed M. Ouf, Francesco Salamone, Marcel Schweiker, Anna Laura Pisello

**Affiliations:** 1 Laboratory of Energy Efficiency in Buildings, Federal University of Santa Catarina, Florianópolis, Brazil; 2 EAPLAB at CIRIAF – Interuniversity Research Center on Pollution and Environment, University of Perugia, Perugia, 06125 Italy; 3 Department of Engineering, University of Perugia, Perugia, PG Italy; 4 Department of Building Services and Process Engineering, Faculty of Mechanical Engineering, Budapest University of Technology and Economics, Budapest, Hungary; 5 Construction Technologies Institute, National Research Council of Italy (ITC-CNR), San Giuliano Milanese, 20098 MI Italy; 6 Controlled, Adaptive and Responsive Environments Laboratory (CARE), Department of Civil and Environmental Engineering, Northwestern University, Evanston, IL USA; 7 Department of Mechanical and Aerospace Engineering, College of Engineering and Computer Science, University of Central Florida, Orlando, Florida USA; 8 Department of Mechanical & Aerospace Engineering, Syracuse University, Syracuse, NY USA; 9 Department of Civil, Environmental, and Architectural Engineering, Worcester Polytechnic Institute, Worcester, Massachusetts 01609 USA; 10 Healthy Living Spaces Lab, Institute for Occupational, Social and Environmental Medicine, Medical Faculty, RWTH Aachen University, Aachen, Germany; 11 Department of Building, Civil and Environmental Engineering, Gina Cody School of Engineering and Computer Science, Concordia University, Montreal, QC Canada; 12 International Centre for Indoor Environment and Energy, Department of Environmental and Resource Engineering, Technical University of Denmark, Kgs, Lyngby, Denmark; 13 Department of Mechatronics, Optics and Engineering Informatics, Faculty of Mechanical Engineering, Budapest University of Technology and Economics, Budapest, Hungary; 14 Chair of Healthy Living Spaces, Faculty of Architecture, RWTH Aachen University, Aachen, Germany

**Keywords:** Psychology and behaviour, Sustainability, Databases

## Abstract

Understanding cross-modal environmental perception is essential for improving occupant well-being and human-centric building design. This paper presents an open-access, multi-site database developed under the IEA-EBC Annex 79 project to test the Hue-Heat Hypothesis (HHH), which hypothesizes that light hue may influence thermal perceptions. The database comprises 543 experimental rounds conducted in eight laboratories across six countries and diverse climate zones, following a shared, rigorously designed protocol. During summer and winter campaigns, participants were exposed to controlled thermal environments and counterbalanced lighting conditions (neutral, reddish, bluish). The database includes detailed metadata on environmental variables, physiological measurements (i.e., heart rate and skin temperature), and self-reported perceptual responses. It also provides standardized technical documentation for each test room, including the detailed experimental protocol and translated survey instruments. All materials are available on the Open Science Framework under the “Multi-site Hue-Heat-Hypothesis Testing” repository. This resource supports research into multi-domain human comfort, enabling analysis of cross-modal and combined effects on human perception and physiological reactions.

## Background & Summary

Understanding how human environmental perception works is essential for enhancing building design quality and efficiency, thereby improving occupants’ well-being while reducing energy consumption. However, the investigation of this topic is complex, as it relies on collecting occupants’ responses, making it susceptible to bias in data interpretation, particularly when the sample size is too small or not representative of the target population. One of the first major attempts to model human thermal perception was made by Fanger in the 1970s, through a series of controlled climate chamber experiments^1^. These studies systematically varied one factor at a time, based on a six-variable human heat balance model that included environmental factors (air temperature, mean radiant temperature, relative humidity, and air velocity) and personal factors (metabolic rate and clothing insulation). The outcome was the development of the Predicted Mean Vote (PMV) model, which estimates the thermal sensation level of a standard person in a given thermal environment.

Fanger’s theory was widely adopted in international standards such as ASHRAE 55^2^ and ISO 7730^3^. However, by the late 20^th^ century, researchers highlighted the PMV model’s limitations, showing a growing interest in the adaptive comfort hypothesis^4^. This approach emphasized the role of contextual factors and occupants’ thermal history in shaping thermal expectations and preferences. To test the adaptive hypothesis, the ASHRAE RP-884 project compiled data from thermal comfort field studies, summing up 21,000 observations in over 160 buildings worldwide^5^. The project examined reported thermal sensation, acceptability, and preference as a function of indoor and outdoor temperature. Notably, the results revealed that occupants in naturally ventilated buildings demonstrated greater tolerance for a wider range of temperatures than those in HVAC-controlled environments, which led to the development of the adaptive thermal comfort model. Moreover, the public availability of the ASHRAE RP-84 database enabled further research by the scientific community. The ASHRAE Global Thermal Comfort Database II project extended the same database to include more recent field study data and significantly expanded the size of the open-source database to more than 100,000 observations^6^.

The adaptive comfort theory also formed the foundation of the EU-funded Smart Controls and Thermal comfort (SCATs) project, which ran from December 1997 to December 2000^7^. Unlike the ASHRAE project, SCATs used a standardized monitoring setup and questionnaire across all study sites, conducting dedicated field studies in 26 buildings across five European countries, involving 840 occupants in total. The project aimed to develop an Adaptive Control Algorithm (ACA) to regulate building temperature setpoints, demonstrating that energy savings could be achieved without compromising perceived thermal comfort. Additionally, the combination of the open-access SCATs and ASHRAE RP-884 databases offered valuable insights into the relationship between climate and indoor comfort^8^. Similarly, the Chinese thermal comfort database was created as an open-access repository by aggregating results from independent field studies conducted over the past two decades in 49 cities spanning five climate zones^9^. Its primary goal was to inform national indoor environment standards and energy codes while providing a comprehensive resource for analyzing occupants’ perceptual responses across diverse climates.

Beyond human comfort, global initiatives have increasingly focused on collecting and disseminating data on occupant behavior in buildings. Diverse building typologies have been monitored across different locations, including a naturally-ventilated office in Germany^10^, 24 offices in the USA^11^, residential dormitories in the USA^12^, a single-family apartment in China^13^, and affordable senior residential buildings in the USA^14^. These studies often include environmental measurements, shedding light on indoor environmental quality and its impact on human behavior. A notable effort in this field, the Global Building Occupant Behavior Database, compiled data from 34 case studies across 15 countries and 10 climate zones^15^. This resource offers valuable insights into occupancy patterns and occupant–building interactions, helping designers, energy modelers, and consultants improve the accuracy of building energy simulations and load forecasts, ultimately narrowing the so-called “performance gap”^16^.

Recently, the multi-domain comfort theory gained attention among researchers as a more comprehensive framework for assessing human environmental responses, emphasizing how simultaneous physical exposures influence different outcomes, from perceptual to behavioral reactions^17^. Despite growing interest, the cross-modal and combined effects that could be incorporated into future standards for enhancing occupant well-being through sustainable design are still under debate between researchers. This lack of consensus is partly due to the generally low quality of experimental design and reporting, which makes it difficult to compare results or perform meta-analyses^18^. In order to overcome such issue, a collective effort was established within the framework of the IEA EBC Annex 79 project^19^ primarily focused on the development of a common experimental protocol to be replicated in multiple laboratories worldwide for testing one of the most controversial hypotheses concerning cross-modal effects, the Hue-Heat-Hypothesis (HHH), which states that thermal responses (specifically, thermal sensation) are affected by light hues, a stimulus typically related to the visual domain^20^. This hypothesis has also attracted international attention due to its potential for energy savings, particularly through adjustments of cooling and heating setpoints^21,22^. Finally, although physiological effects related to the HHH have been discussed in the literature^23–25^, an open-source database to support such assessments is still lacking.

The present contribution introduces the open-access database on HHH testing derived from a coordinated multi-site controlled experimental study. The database is publicly available within the “Multi-site Hue-Heat-Hypothesis Testing” on the Open Science Framework (OSF) platform. It consists of 24 datasets collected referring to 543 experimental rounds carried out in eight institutions located in six countries and belonging to different climate classes. The OSF project also publishes research materials and the shared experimental protocol. Specifically, each test environment is thoroughly documented using a standardized technical sheet, and detailed information is provided regarding experimental conditions, participants’ recruitment criteria, and study design. These comprehensive metadata support investigations of the HHH and broader research on cross-modal and multi-domain environmental effects, encompassing both perceptual and physiological responses. Moreover, the procedure is designed and reported to be replicable, allowing additional research groups to contribute new datasets and strengthening the statistical power and generalizability of related analyses.

## Methods

This section introduces the experimental protocol and the research institutes participating in this project.

### Participants

Each laboratory defined its own strategy for recruiting participants. All subjects provided informed consent before participating in the experiment. When ethics approval from a specific review board was required by the institution or government, each laboratory was responsible for obtaining it. The protocol recommended that participants in the experimental rounds should be adults aged between 18 and 40 years, with an even balance by gender at each research institute, to guarantee consistent standards for the experiment. The final database includes, in total, data from 543 experimental rounds with 52.7% male and 46.9% female participants. One participant involved in two experimental rounds preferred not to disclose their gender. In addition, participants taking part in seven other experimental rounds were older than 40 years. Despite these deviations from the protocol, representing a minor number of cases, these rounds were included in the final database. Some laboratories required participants to wear specific garments to ensure the same clothing level for each season, i.e., 0.5 clo in summer and 1.0 clo in winter. All participants’ personal information, including clothing, was requested in the initial general survey (detailed in the Materials subsection) so that the clothing insulation (defined by summing the insulation of individual garments based on the ISO 7730^3^) was reported in the database for all participants, even for laboratories without specific garment instructions.

### Experimental protocol

To challenge the HHH, the experiments focused on the exposure of recruited subjects to specific combinations of thermal and visual stimuli during summer and winter rounds, as synthesized in Table 1. Air temperature, air velocity, air humidity, and globe temperature were measured every minute in the vicinity of the participant (about 30 cm). Specifically, air temperature and velocity were measured at the ankle and head levels, i.e., 10 and 110 cm height, to verify the assumption of a homogeneous thermal environment and avoid local discomfort. Moreover, 5 out of 8 laboratories included a detailed characterization of the Spectral Power Distribution of each lighting condition experienced by participants. These measurements are detailed in the technical sheets for each test room, which are publicly available in the OSF repository under the Materials and Methods section. Since the experiments did not allow for daylighting, which is intrinsically dynamic by nature, visual measurements could be done at once before the experiments for each lighting condition (neutral, reddish, and bluish).

**Table 1:** Conditions adopted for thermal and visual domains.

Domain	Factors	Levels			
Thermal	Air temperature [°C]	Cold		Hot	
		20^a^	24^a^	26^b^	28^b^
	Air velocity	Constant (<0.1 m/s)			
Visual	Light CCT [K]	Reddish	Neutral	Bluish	
		2700–3000	~4000	6000–6500	
	Illuminance	~500 lx (on the desk, horizontal plane)			
		~300 lx (at the sight level, vertical plane)			

(^a^temperatures for winter experiments; ^b^temperatures for summer experiments).

Additionally, physiological measurements comprised skin temperatures and heart rates. A 10-point method was adopted for the skin temperatures due to its reliability and sensitivity^26^. In detail, the points for skin temperature monitoring were: forehead, right upper arm, left forearm, right hand, left back, left chest, left abdomen, anterior thigh, anterior calf, and right foot. Heart rates were measured with wrist-worn devices due to reduced invasiveness and relatively high accuracy under short-term campaigns with reduced activity levels of the subject^27^. The experimental protocol did not require metabolic rate monitoring, but it was designed to keep it around 1 met (58 W/m²), corresponding to seated, relaxed activities^3^.

**Fig. 1:**
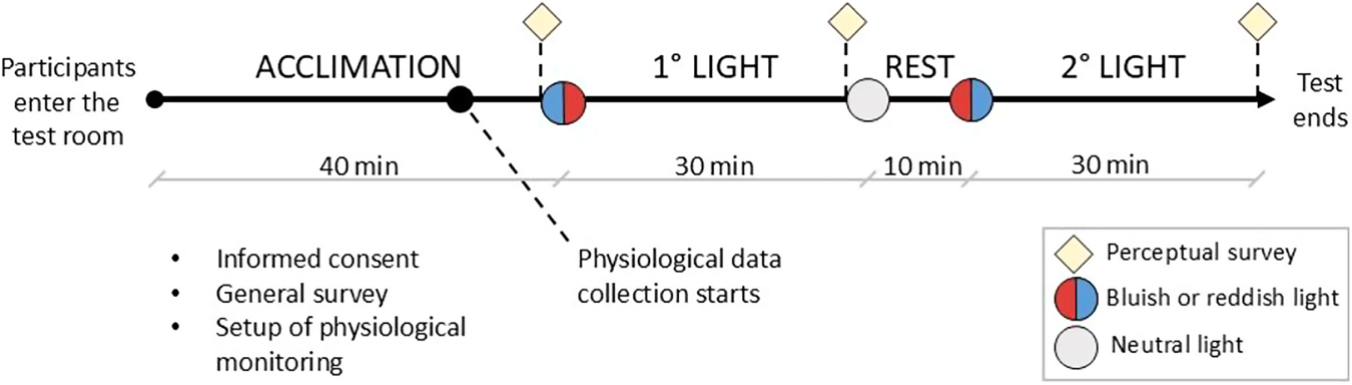
Overview of experiment procedure. Each experiment was repeated two times in each season with fixed thermal conditions (24 °C for Summer/Cool, 28 °C for Summer/Warm, 20 °C for Winter/Cool, and 26 °C for Winter/Warm) presented in a counterbalanced order.

Each experiment lasted 110 minutes (Fig. 1), conducted under fixed thermal conditions (24 °C for Summer/Cool, 28 °C for Summer/Warm, 20 °C for Winter/Cool, and 26 °C for Winter/Warm) and changing lighting conditions presented in a counterbalanced order to ensure the high quality of the experiment. For instance, half of the participants were exposed to the low CCT (reddish) lighting as the first condition, while the rest were exposed to the high CCT (bluish) lighting as the first condition. Following a repeated measures design, with thermal conditions being repeated, each subject participated twice on two non-consecutive days to guarantee at least one wash-out day between the two rounds. All the tests were performed in the morning to keep the influence of the circadian rhythm comparable between the participants. Participants remained engaged in sedentary office tasks (seated, reading, or writing), representing a metabolic rate of 1.0 met. The experiments were divided into the following stages:

- **Acclimation**: The initial 40 minutes^28^ of the experiment consisted of subject preparation and acclimation to the thermal condition while exposed to neutral lighting (around 4000 K^22^). During the first 30 minutes, the participants received explanations about the experimental procedure. They were asked to read an information sheet, sign a consent form, and complete a survey with their characteristics and general preferences. Additionally, wearable sensing technologies for physiological monitoring were applied within this timeframe to guarantee a record of at least 10 minutes of physiological data at the end of the acclimation period.
- **First lighting condition**: During the following 30 minutes, participants were exposed to the first lighting condition. The duration was defined according to previous studies where people were exposed to different lighting conditions^29,30^. After such exposure, they filled standard right-here-right-now thermal and visual perception surveys.
- **Resting period**: Between the first and the second lighting conditions, participants were exposed to neutral lighting for 10 minutes to minimize carry-over effects^29–31^. During this phase, participants were asked to do gentle movements (i.e., on-site walking for 5 minutes) to avoid otherwise decreasing metabolic rate due to prolonged sitting and keep it at a comparable level to the previous ones.
- **Second lighting condition**: In the last 30 minutes, participants were exposed to the second lighting condition and, in the end, answered the same right-here-right-now questionnaire.

### Experimental facilities

This project involved efforts from eight research institutes worldwide, as listed in Table 2. Such a vast group of research institutes also resulted in varied characteristics of the facilities regarding geometric characteristics and systems available. Each group provided a technical sheet of their facility with detailed descriptions as part of the activity, available on the OSF platform for in-depth evaluation^32^. Table 3 synthesizes the main characteristics of all the facilities.

**Table 2:** Research Institutes joining the interlaboratory experimental procedure.

TR	Research Institute (acronym)	Location (city, country)	Köppen-GeigerClimate Class^33^	ASHRAE climate zone^34^
1	Worcester Polytechnic Institute (WPI)	Worcester, USA	Dfb	5 A
2	Construction Technologies Institute (ITC)	Milan, Italy	Cfa	4 A
3	RWTH Aachen University (RWTH)	Aachen, Germany	Cfb	5 A
4	Syracuse University (SU)	Syracuse, USA	Dfb	5 A
5	Budapest University of Technology and Economics (BME)	Budapest, Hungary	Cfa	5 A
6	Federal University of Santa Catarina (UFSC)	Florianópolis, Brazil	Cfa	2 A
7	Concordia University (CU)	Montreal, Canada	Dfb	6 A
8	University of Perugia (UNIPG)	Perugia, Italy	Cfa	4 A

**Table 3:** Synthesis of the main characteristics of each test room involved in this study.

TR	Dimensions	Boundary conditions	Finishing	Conditioning system(s)	Lighting system(s)
1	4.60 m × 5.90 m × 3.60 m	Room in a building	Walls: white paint; floor: cool grey cover; ceiling: coated with steel fireproof spray	HVAC system (centralized air conditioning, ceiling-mounted heater, and fans)	4 suspended LED lights with CCT control
2	6.40 m × 3.70 m × 2.95 m	Room in a building	Walls: plaster, white paint; floor: dark grey steel tiles with satin finish and calamine texture; ceiling: white soundproof tiles	Radiant system (radiant floor modules for heating and cooling), HVAC system (heat pump with recovery systems)	1 suspended LED panel with CCT control, 1 LED lamp, 1 halogen lamp
3	3.00 m × 4.00 m × 2.55 m	Room in a building	Walls: white coloured punched metallic boards (thermal radiators), white painted plasterboards, black-colored metal strips dividing these surfaces; ceiling: white coloured punched metallic boards; floor: dark grey vinyl flooring.	Radiant system (15 heating and cooling panels distributed on the ceiling and right and left walls), HVAC system (heat supply through district heating system, cooling supply through air-to-water heat pump, humidification through steam humidifier)	2 free-standing LED panels with direct and indirect light output (2700–6000 K), 1 LED strip with tunable white light and RGB colors (2300–6500 K)
4	10.97 m × 5.10 m × 3.20 m	Room in a building	Walls: white painting; floor: fabric carpet; ceiling: white partitions	HVAC system (mixed and displacement ventilation)	4 panel luminaires, LED desk lamps with CCT control
5	4 m × 4 m × 3 m	Independent volume inside a building	Walls: 3 white-painted, 1 black-painted (enables visualizing airflows); floor: floor tiles; ceiling: cassette type	Radiant system (panels on the walls, floor, and ceiling), HVAC system (compact air handling unit)	4 LED bulbs (2700 K), 4 classic LED bulbs (6500 K), 2 + 2 LED bulbs (4000 K), 12 LED fixture + RGB LED reflectors
6	2.80 m × 3.50 m × 2.62 m	Independent volume inside a building	Walls and ceiling: gypsum board, white painting; floor: light brown vinyl flooring	HVAC system (Variable Refrigerant Flow system)	4 LED panels with CCT control
7	2.8 m × 3.8 m × 2.7 m	Independent volume inside a building	Walls: polished chrome plasterboard; floor: grey; ceiling: suspended panels	Perimeter heating system (2 convectors with high heat output), HVAC system (Air Handling Unit + Variable Air Volume system)	4 LED panels, RGB reflectors
8	4.0 m × 4.0 m × 2.7 m	Independent volume inside a building	Walls, floor, and ceiling: grey plasterboard; dark grey strip in the middle of the walls for visualizing the radiant system	Radiant system (heating/cooling in all surfaces), HVAC system (air-to-air heat pump, active heat recovery)	4 LED panels, 2 RGB reflectors (14 emission colors)

### Materials

The materials used in the experimental sessions included two distinct surveys (general and perceptual) and the sensors to record environmental and physiological parameters (monitored variables described in the Experimental protocol subsection). The experimental protocol specified only that these parameters must be provided at 1-minute intervals, allowing each laboratory to use its own set of sensors. Sensor specifications (name and accuracy) for each laboratory are detailed in their respective technical sheets, which are available in the OSF repository^32^.

The general survey, completed at the beginning of each experimental round, gathered information on participants’ clothing, age, gender, height, weight, highest level of education, employment status, visual problems, and in which city they were currently living and for how long (subjects who had lived in the same city for less than three consecutive years were also asked to specify their previous place of residence). Participants also provided information about their sleep quality (5-point Likert-like scale from 1 - worst - to 5 - best), stress level (5-point Likert-like scale from 1 - worst - to 5 - best), and eating and exercise habits (5-point scale from 1 - uncommon - to 5 - regular) over the past seven days. The initial survey concluded with questions related to comfort sensitivity, where participants rated their self-perceived sensitivity to cold climates, hot climates, glare, bright light exposure, insufficient light, and poor air circulation (answers on a 5-point Likert-like scale from 1 - lowest - to 5 - highest). Finally, at the end of the acclimation period (neutral lighting) and reddish and bluish light exposures, they answered a right-here-right-now perceptual survey including questions about thermal and visual sensation, comfort, preference, and acceptability, as well as thermal sensation at the hands, trunk, and feet, and overall comfort. Responses were recorded using standardized scales: sensations and preferences were rated on 7-point Likert-like scales ranging from 1 (cold/too dark; much cooler/darker) to 7 (hot/too bright; much warmer/brighter); comfort was measured on an intensity scale ranging from 1 (comfortable) to 5 (extremely uncomfortable); and acceptability was measured on a symmetrical 4-point scale from 1 (clearly acceptable) to 4 (clearly unacceptable), without a neutral option. The general and perceptual surveys used in this experimental protocol are included in the OSF repository^32^ in English. Some laboratories also made available their translations in their official languages.

### Ethics and consent

Ethical approval was obtained from the relevant institutional or national review board when required (WPI: Institutional Review Board (IRB-20–0001); ITC: CNR Ethics and Research Integrity Commission (0053590/2022); RWTH: The Ethics Committee at the RWTH Aachen University Faculty of Medicine (EK 23-046); SU: Institutional Review Board (22–161); BME: United Ethical Review Committee for Research in Psychology (2022-123); UFSC: Research Ethics Committee Involving Human Subjects (5.993.216); CU: Human Research Ethics Committee (30016771)). These boards approved the content of the consent form, which was signed by all the participants involved in the experiments provided in the OSF database.

## Data Records

The datasets are publicly available on the OSF project page^32^ and can be accessed without an OSF account. The data were licensed under a CC-By Attribution 4.0 International license. The datasets are provided in comma-separated values (.csv) format, including environmental and physiological parameters monitored during the experimental rounds, subjective responses of participants collected during the tests, and outdoor weather conditions. Each file is named systematically using an identifier for the test room (TR from Tables 1, 2) and additional descriptors to clarify the content, as follows:

- “env_physiological” for the indoor environment measurements and physiological signals taken during all rounds;
- “questionnaire” for the files with subjective answers;
- “outdoor_weather” for weather data.

For example, file names include “env_physiological_lab8.csv” as indoor and physiological measurements for test room 8 for all the carried out experimental rounds; “questionnaire_lab3.csv” as the subjective responses collected in test room 3 for all participants; and “outdoor_weather_lab7.csv” as the outdoor conditions measured close to test room 7. Table 4 synthesizes the data presented in each file. In addition, the OSF project documentation provides a comprehensive guideline detailing the dataset structure and naming conventions.

**Table 4:** Dataset description.

Code	File: “env_physiological”	Type	Format	Unit
ID_full	Test ID	Identifier	lab_season_condition_order-of-lighting_subjectID	—
TS	Timestamp of the record	Temporal	DD/MM/YYYY HH:MM	—
Lc	Lighting condition	Categorical	n: Neutral; r: Reddish; b: Bluish.	—
Ta	Air temperature at 110 cm	Continuous	Float	°C
Tank	Air temperature at 10 cm	Continuous	Float	°C
Rh	Relative humidity	Continuous	Float	%
MRT	Mean radiant temperature	Continuous	Float	°C
Va	Air velocity at 110 cm	Continuous	Float	m/s
Vank	Air velocity at 10 cm	Continuous	Float	m/s
Eh	Illuminance on the horizontal plane (on the desk)	Continuous	Float	lx
Ev	Illuminance on the vertical plane (at the sight level, 120 cm)	Continuous	Float	lx
CCTh	CCT on the horizontal plane (on the desk)	Continuous	Integer	K
CCTv	CCT on the vertical plane (at the sight level, 120 cm)	Continuous	Integer	K
Tsk_A	Skin temperature at the head	Continuous	Float	°C
Tsk_D	Skin temperature at the shoulder	Continuous	Float	°C
Tsk_F	Skin temperature at the forearm	Continuous	Float	°C
Tsk_H	Skin temperature on the hand	Continuous	Float	°C
Tsk_J	Skin temperature at the back	Continuous	Float	°C
Tsk_K	Skin temperature at the chest	Continuous	Float	°C
Tsk_M	Skin temperature at the abdomen	Continuous	Float	°C
Tsk_O	Skin temperature at the thigh	Continuous	Float	°C
Tsk_Q	Skin temperature at the shin	Continuous	Float	°C
Tsk_T	Skin temperature at the foot	Continuous	Float	°C
HRinst	Instantaneous heart rate	Continuous	Float	bpm
HRave	Average heart rate	Continuous	Float	bpm
**Code**	**File: “questionnaire”**	**Type**	**Format**	**Unit**
ID_full	Test ID	Identifier	lab_season_condition_order-of-lighting_subjectID	—
Date	Date of the test	Temporal	DD/MM/YYYY	—
Iclo	Clothing insulation level	Continuous	Float	clo
Lc	Lighting condition	Categorical	n: Neutral; r: Reddish; b: Bluish.	—
DQ1	Age	Categorical	a: Under 21; b: 21–25; c: 26–35; d: 36–40; e: 40–55; f: Over 55.	—
DQ2	Gender	Categorical	a: Male; b: Female; c: I do not want to answer.	—
DG3	Level of education	Categorical	a: Less than a high school diploma; b: High school or equivalent degree; c: Bachelor’s degree; d: Master’s degree; e: PhD or higher; f: None; g: I do not want to answer.	—
DQ4	Employment status	Categorical	a: Employed full-time (40 + a week); b: Employed part-time (less than 40 hours a week); c: Unemployed (currently looking for a job); d: Unemployed (not currently looking for a job); e: Student; f: Retired; g: Self-employed.	—
DQ5	Height	Continuous	Float	m
DQ6	Weight	Continuous	Integer	kg
DQ7	Visual problems (if any)	String	—	—
DQ8	Country and city of residence	String	—	—
DQ9	Period living in this city	Categorical	a: <1 year; b: 1–3 years; c: >1 year.	—
DQ10	If this period is less than three consecutive years, the country(ies) and city(ies) of residence during the past 3 years	String	—	—
BQ1	Sleep quality rate in the past seven days	Ordinal	1, 2, 3, 4, or 5	—
BQ2	Stress level in the past seven days	Ordinal	1, 2, 3, 4, or 5	—
BQ3	Eating habits in the past seven days	Ordinal	1, 2, 3, 4, or 5	—
BQ4	Exercise habits in the past seven days	Ordinal	1, 2, 3, 4, or 5	—
SQ1	Sensitivity to cold climates	Ordinal	1, 2, 3, 4, or 5	—
SQ2	Sensitivity to hot climates	Ordinal	1, 2, 3, 4, or 5	—
SQ3	Sensitivity to glare	Ordinal	1, 2, 3, 4, or 5	—
SQ4	Sensitivity to bright light exposure	Ordinal	1, 2, 3, 4, or 5	—
SQ5	Sensitivity to insufficient light	Ordinal	1, 2, 3, 4, or 5	—
SQ6	Sensitivity to poor air circulation	Ordinal	1, 2, 3, 4, or 5	—
PQ1	Thermal sensation (for neutral, bluish, and reddish lighting conditions)	Ordinal	1, 2, 3, 4, 5, 6, or 7	—
PQ2	Thermal comfort (for neutral, bluish, and reddish lighting conditions)	Ordinal	1, 2, 3, 4, or 5	—
PQ3	Thermal preference (for neutral, bluish, and reddish lighting conditions)	Ordinal	1, 2, 3, 4, 5, 6, or 7	—
PQ4	Hands thermal sensation (for neutral, bluish, and reddish lighting conditions)	Ordinal	1, 2, 3, 4, 5, 6, or 7	—
PQ5	Trunk thermal sensation (for neutral, bluish, and reddish lighting conditions)	Ordinal	1, 2, 3, 4, 5, 6, or 7	—
PQ6	Feet thermal sensation (for neutral, bluish, and reddish lighting conditions)	Ordinal	1, 2, 3, 4, 5, 6, or 7	—
PQ7	Thermal acceptability (for neutral, bluish, and reddish lighting conditions)	Ordinal	1, 2, 3, or 4	—
PQ8	Visual sensation (for neutral, bluish, and reddish lighting conditions)	Ordinal	1, 2, 3, 4, 5, 6, or 7	—
PQ9	Visual comfort (for neutral, bluish, and reddish lighting conditions)	Ordinal	1, 2, 3, 4, or 5	—
PQ10	Visual preference (for neutral, bluish, and reddish lighting conditions)	Ordinal	1, 2, 3, 4, 5, 6, or 7	—
PQ11	Visual acceptability (for neutral, bluish, and reddish lighting conditions)	Ordinal	1, 2, 3, or 4	—
PQ12	Overall comfort (for neutral, bluish, and reddish lighting conditions)	Ordinal	1, 2, 3, 4, or 5	—
**Code**	**File: “outdoor_weather”**	**Type**	**Format**	**Unit**
TS	Timestamp of the data	Temporal	DD/MM/YYYY HH:MM	—
S	Season in which the measurement was taken	Categorical	s: Summer; w: Winter.	—
Ta	Air temperature	Continuous	Float	°C
Lat	Latitude of the weather station	Continuous	Float	°
Lon	Longitude of the weather station	Continuous	Float	°

Both “env_physiological” and “questionnaire” files associate each experimental round to a unique identifier (“ID”), which represents the combination of the laboratory number (TR in Tables 1, 2), season (“s” for summer and “w” and winter), thermal condition (“c” or “h” denoting cold or hot round), the lighting sequence (“br” for bluish–reddish or “rb” for reddish–bluish), and participant gender (“F” for female, “M” for male, or “X” for participants who chose not to disclose their gender), followed by an identifier number for each participant. For example, “8_w_h_br_F11” indicates a test in test room 8 in winter, during a hot round with bluish lighting first, recorded for participant F11, who was a female. The ID number enables the association of environmental and physiological measurements in the file “env_physiological” with subjective perceptions in the file “questionnaire” during a specific round.

### Repository organization and contents

The OSF project is separated into two main components. The first one (named “Materials and Methods”) includes three folders: one named “Experimental Protocol” with a file specifying the procedures for carrying out the experiment and the details for data organization; one with the technical specifications for each contributing laboratory (“Technical sheets”), with one file per laboratory; and one (“Questionnaires”) holding the questionnaires in different languages, including English.

The second main component (“Data”) stores the datasets. For each contributing laboratory, there is a separate folder containing the three files previously described (“env_physiological”, “questionnaire”, and “outdoor_weather”). Data within these files are categorized according to the experiment’s season.

## Technical Validation

Technical validation was performed to ensure the reasonability of data and consistency with the proposed experimental protocol. This involved an initial analysis of the data provided by each laboratory to verify the uniformity of units for objective data and scales for subjective data, as informed in the protocol and data organization documentation available on the OSF platform.

Afterwards, the environmental data collected during the experimental rounds were validated to verify their coherence, with flexibility to account for the different backgrounds and system particularities of each laboratory involved in the initial experiments. Therefore, specific quality criteria thresholds were defined. In detail, a valid experimental round should present air temperature (mean of measurements at ankle and head levels) between 18 °C and 30 °C, relative humidity between 10% and 80%, air velocity (mean of measurements at head and ankle levels) below 1 m/s, and illuminance on the horizontal plane above 200 lx. Air temperature values were used to approximate mean radiant temperature for rounds where the latter was not recorded. The final database did not include any experimental round with at least one record out of these limits. Moreover, rounds where no environmental data related to the thermal domain (i.e., air temperature, mean radiant temperature, relative humidity, and air velocity) were recorded due to technical problems were not included. After this technical validation, 543 experimental rounds (90% of all carried out rounds) were included in the database.

### Environmental variables and clothing levels

**Fig. 2:**
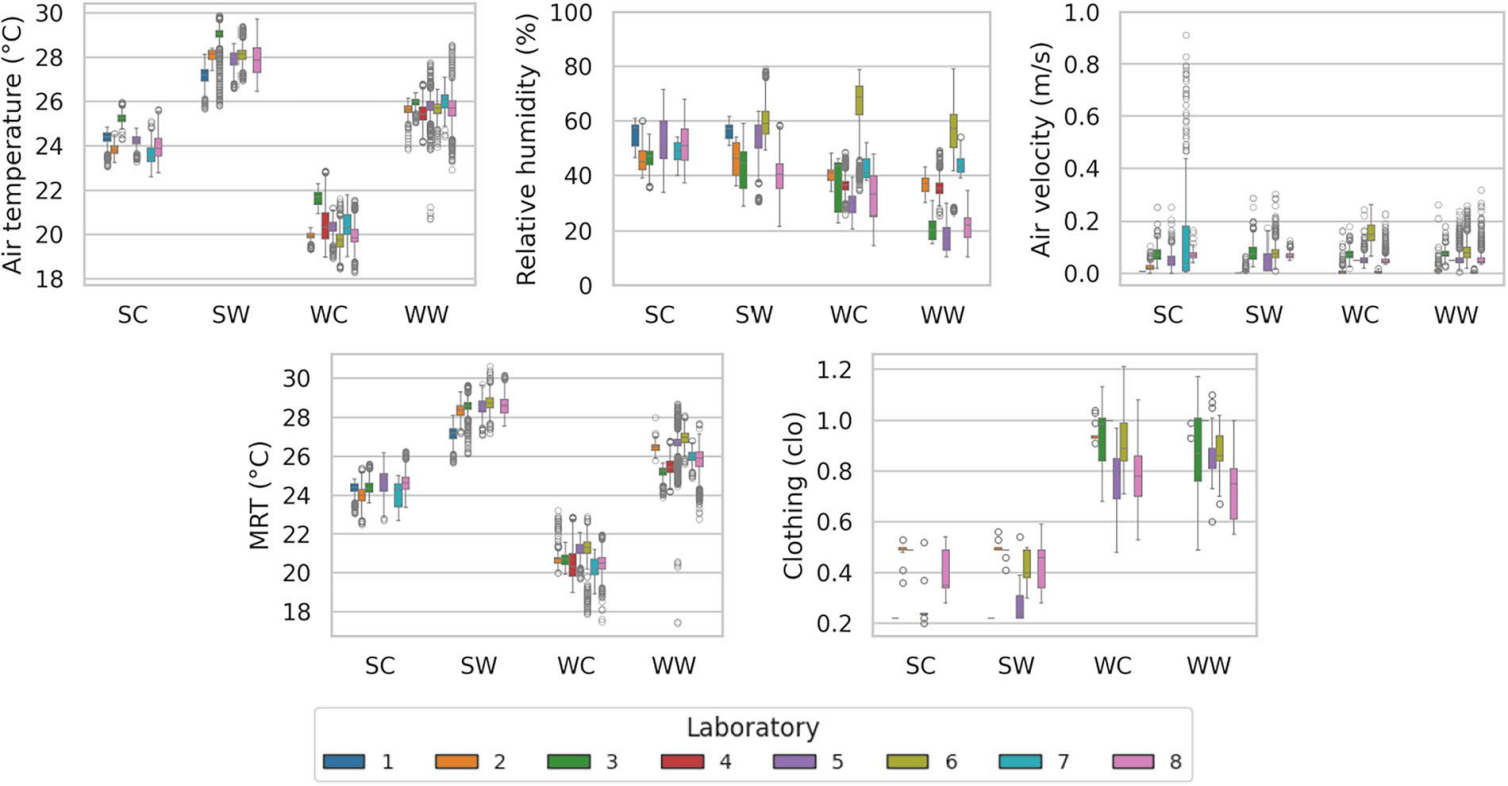
Environmental variables and clothing levels during the experiments.

Figure 2 presents the distribution of key environmental variables and participants’ clothing levels throughout the tests. Such variables (along with those presented in subsequent sections) were stratified according to the experimental conditions: SC (Summer/Cool), SW (Summer/Warm), WC (Winter/Cool), and WW (Winter/Warm). The variables were measured or estimated according to the criteria in the experimental protocol. While thermal variables cluster around the expected thresholds for each experimental condition, variability across laboratories reflects environmental control strategies of each TR and local climatic characteristics. Such inter-laboratory differences are explicitly documented in the metadata available in the OSF repository, enabling future cross-study analyses and replication.

### Physiological signals

**Fig. 3:**
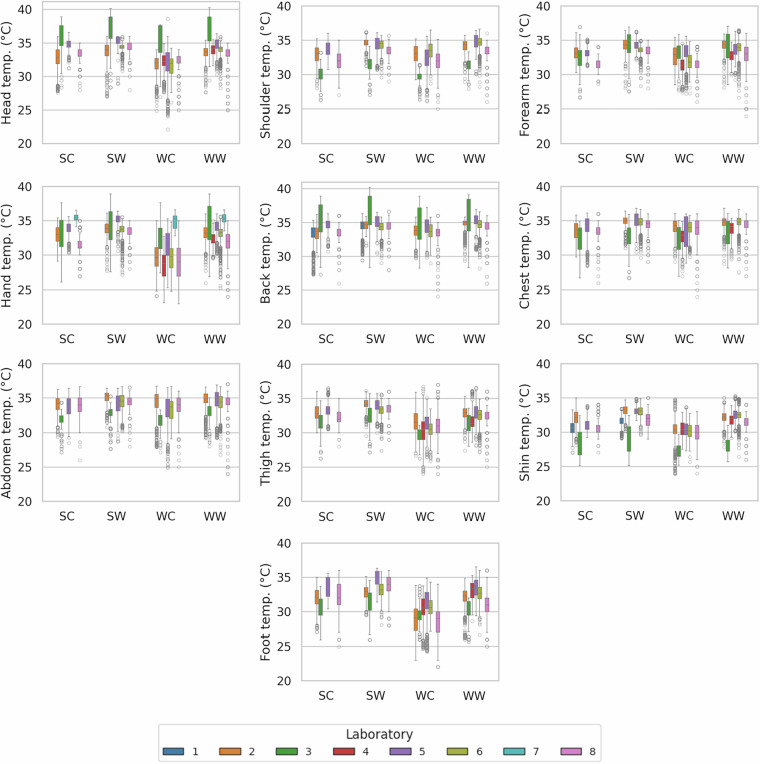
Skin temperatures measured over ten body locations.

**Fig. 4:**
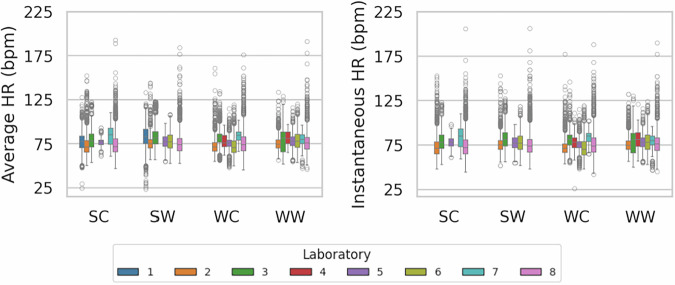
Average and instantaneous Heart Rates.

Skin temperatures were measured at ten body locations (Fig. 3), and instantaneous and average heart rates were recorded (Fig. 4). Although minor instrumentation differences existed among laboratories, physiological signals remained within expected ranges according to seasonal variations. Core-to-peripheral skin temperature gradients were observed: lower temperatures and higher variability at distal sites (e.g., hands) than central areas (e.g., chest, abdomen). Heart rate values ranged from 50 to 150 bpm, consistent with physiologically plausible limits. Outliers and high variability in physiological signals provide opportunities to investigate individual differences in response to indoor thermal conditions. Publicizing the full database allows subsequent researchers to apply customized filters or thresholds for further hypothesis testing.

### Multi-domain human perceptions

**Fig. 5:**
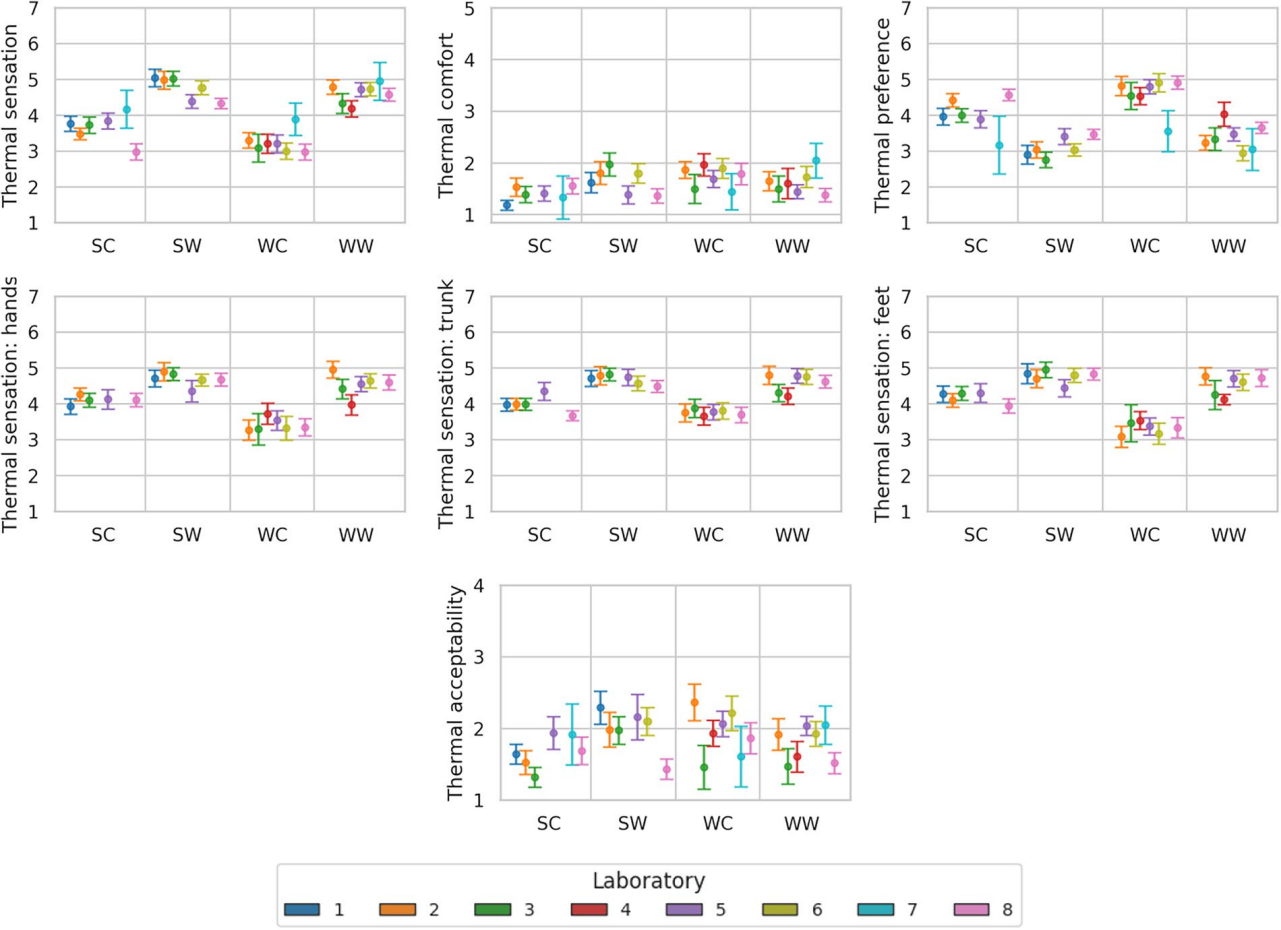
Thermal perceptions reported by the participants.

**Fig. 6:**
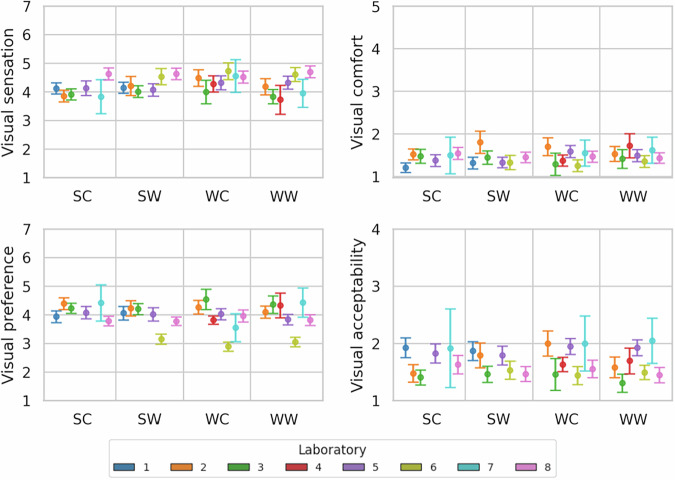
Visual perceptions reported by the participants.

**Fig. 7:**
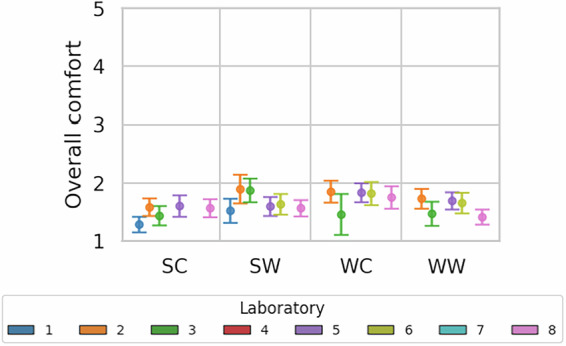
Overall comfort reported by the participants.

Thermal perceptions are reported in Fig. 5. In general, thermal sensations, comfort, and preference are consistent with the expected outcomes for each experimental condition defined in the protocol. Inter-laboratory agreement confirms the reproducibility of the experimental protocol, and relatively low error margins enhance the credibility of the database. Greater variability was observed for thermal acceptability, opening the room for further analyses, including comparisons with field studies (e.g., ASHRAE global thermal comfort database). Thermal acceptability was rated using the smallest scale, which may have contributed to the variations. Such in-depth evaluation of thermal perceptions complements physiological measurements, reinforcing the multidimensional contribution of this database. Importantly, although the human perceptions represented in Figs. 5–7 are ordinal in nature, the figures present means and standard deviations to improve readability.

Figure 6 displays the consistency of visual perception responses collected during the experimental rounds. Despite architectural, geographical, and operational differences across laboratories, visual perceptions were largely consistent, indicating good inter-laboratory comparability. Similar to the thermal domain, visual acceptability was measured using the smallest scale compared to the other variables, possibly influencing its distribution.

In addition to single-domain perceptions, Fig. 7 illustrates the overall comfort across laboratories and experimental conditions. While consistent patterns were observed in most laboratories, the outcomes of laboratory 4 exhibited substantially higher mean scores. Despite this outlier, the tight confidence intervals suggest good consistency.

## Usage Notes

The database has been uploaded to a public OSF repository^32^ and can be downloaded without an OSF account. Users will find several .csv files available, including:

1. Full datasets per laboratory, containing environmental variables and participants’ psychophysiological responses;
2. Datasets with only environmental and physiological variables;
3. Datasets with questionnaire responses;
4. Files with outdoor weather conditions.

As previously mentioned, 49 experimental rounds were excluded during technical validation and are not included in the final database. No additional exclusion criteria were applied, allowing future users to define their inclusion parameters based on specific research goals.

## Data Availability

The datasets are publicly available on the OSF project page^32^ (https://osf.io/sdb7q/) and can be accessed without an OSF account.

## Code availability

This study did not involve using any custom code in the generation or processing of the database. Data cleaning and visualization were performed using standard Python libraries.
